# The influence of upright posture on craniospinal, arteriovenous, and abdominal pressures in a chronic ovine in-vivo trial

**DOI:** 10.1186/s12987-023-00485-6

**Published:** 2023-11-09

**Authors:** Anthony Podgoršak, Nina Eva Trimmel, Markus Florian Oertel, Margarete Arras, Miriam Weisskopf, Marianne Schmid Daners

**Affiliations:** 1 Department of Mechanical and Process Engineering, ETH Zurich, Zurich, Switzerland; 2 Center for Preclinical Development, University Hospital Zurich, University of Zurich, Zurich, Switzerland; 3 Department of Neurosurgery, University Hospital Zurich, University of Zurich, Zurich, Switzerland

**Keywords:** Arterial blood pressure, Cerebrospinal fluid, Hydrocephalus, Intracranial pressure, Ovine model, Physiology

## Abstract

**Introduction:**

Most investigations into postural influences on craniospinal and adjacent physiology have been performed in anesthetized animals. A comprehensive study evaluating these physiologies while awake has yet been completed.

**Methods:**

Six awake sheep had telemetric pressure sensors (100 Hz) implanted to measure intracranial, intrathecal, arterial, central venous, cranial, caudal, dorsal, and ventral intra-abdominal pressure (ICP, ITP, ABP, CVP, IAPcr, IAPcd, IAPds, IAPve, respectively). They were maneuvered upright by placing in a chair for two minutes; repeated 25 times over one month. Changes in mean and pulse pressure were calculated by comparing pre-chair, P0, with three phases during the maneuver: P1, chair entrance; P2, chair halftime; P3, prior to chair exit. Statistical significance (p ≤ .05) was assessed using repeated measures ANOVA.

**Results:**

Significant mean pressure changes of (P1 − P0) and (P3 − P0) were measured at − 12.1 ± 3.1 and − 14.2 ± 3.0(*p* < .001), 40.8 ± 10.5 and 37.7 ± 3.5(*p = .019*), 9.7 ± 8.3 and 6.2 ± 5.3(*p = .012*), 22.3 ± 29.8 and 12.5 ± 12.1(*p = .042*), and 11.7 ± 3.9 and 9.0 ± 5.2(*p = .014*) mmHg, for ICP, ITP, IAPds, IAPcr, IAPca, respectively. For pulse pressures, significant changes of (P1 − P0) and (P3 − P0) were measured at − 1.3 ± 0.7 and − 2.0 ± 1.1(*p* < .001), 4.7 ± 2.3 and 1.4 ± 1.4(*p* < .001), 15.0 ± 10.2 and 7.3 ± 5.5(*p* < .001), − 0.7 ± 1.8 and − 1.7 ± 1.7(*p* < .001), − 1.3 ± 4.2 and − 1.4 ± 4.7(*p =* .006), and 0.3 ± 3.9 and − 1.0 ± 1.3(*p* < .001) mmHg, for ICP, ITP, ABP, IAPds, IAPcr, IAPca, respectively.

**Conclusions:**

Pressures changed posture-dependently to differing extents. Changes were most pronounced immediately after entering upright posture (P1) and became less prominent over the chair duration (P2-to-P3), suggesting increased physiologic compensation. Dynamic changes in IAP varied across abdominal locations, motivating the abdominal cavity not to be considered as a unified entity, but sub-compartments with individual dynamics.

**Supplementary Information:**

The online version contains supplementary material available at 10.1186/s12987-023-00485-6.

## Introduction

Hydrocephalus is a neurological disease characterized by disturbed cerebrospinal fluid (CSF) dynamics [1]. Its treatment has been a topic of debate ever since the first documented use of a valved CSF shunt around 70 years ago [2]. The ability to design more sophisticated treatment options to treat CSF-related disorders is truly limited until gaps in quantitative understanding of CSF dynamics and their communication to the adjacent compartments are filled. Quantifying the interactions between intracranial pressure (ICP), intrathecal pressure (ITP), arterial blood pressure (ABP), central venous pressure (CVP), and abdominal pressures (IAP) whilst experiencing prone-to-standing postural changes can help elucidate the dynamic relationships that exist between these unique compartments, both improving our understanding of the underlying physiology as well as pave the way for more sophisticated treatment options.

There have been many previous studies that have helped form the foundation of our physiologic understanding of the impact of posture on CSF and its interplay between other anatomical compartments. Tilt tests (TTs) have been ubiquitously used in clinical and research circles since 1986, when the head-up tilt was first described as a useful non-invasive tool to investigate unexplained syncope [3]. Usually comprised of the exposure of a subject to controlled orthostatic changes in a safe, monitored, laboratory environment, TTs are still used today to replicate symptoms and induce autonomic regulatory changes via manipulations to body position that would otherwise be impossible [4, 5].

Body position’s influence on physiologic factors has been well documented within the literature, with the effect of posture on oxygen transport being reported as early as the 1960s [6, 7]. Cranially, body position has been heavily reported to influence ICP via the altered cranial-caudal hydrostatic gradient and volumetric redistributions of CSF [8]. Also, it can influence functional connectivity through changes in relative heart location leading to a decrease in vagal activity which in return increases sympathetic activity in addition to intracranial compliance via volumetric redistributions of CSF [9, 10]. Body position has further shown to influence cerebral blood flow by changing cerebral arterial and venous morphology [11], and glymphatic transport due to changes in CSF efflux patterns from physical anatomical changes [12], among others. Extracranially, it is known that body position heavily influences arterial and venous function in addition to respiratory response [13–15].

The use of sheep as models to study CSF-related physiologies has been well documented [16–23]. Specific examples of sheep models for CSF related investigation include the work of Di Rocco et al. [17, 18], Cambria et al. [19], and more recently Oria et al. [20] and Emery et al. [21], including previous work from the authors [16, 22–24]. Moreover, the sheep used in this study were of the White Alpine variety, making the upright postures closer to their natural environment when compared to other flatter-land sheep breeds. However, previous studies have all been performed in controlled, laboratory environments, with studies being done under general anaesthesia. Due to the influence of anesthetic agents, pressure dynamics and autonomic compensatory mechanisms are different than in awake sheep [25]. This supports the need for chronic, awake investigations due to their reduction of external confounding effects on pressure dynamics. Furthermore, the influence of posture on physiologic pressures in awake, free-roaming subjects is far less studied. In fact, to date, a study that simultaneously and comprehensively measures various unique pressures known to influence CSF dynamics in a chronic setting has yet been accomplished. In our chronic in-vivo ovine study, the intracranial, intrathecal, arterial, central venous, and abdominal pressures are measured simultaneously while performing a postural change by maneuvering the sheep into a sheep chair. These multi-compartmental pressures are interrelated and quantified to provide insights into the physiologic reactions under gravitationally induced dynamic changes.

## Methods

### Ethical statement

Animal housing and all experimental procedures were approved by the local Committee for Experimental Animal Research (Cantonal Veterinary Office Zurich, Switzerland) under the license number ZH119/2019, and were conforming to the European Directive 2010/63/EU of the European Parliament and the Council on the Protection of Animals used for Scientific Purposes, as well as to the Guide for the Care and Use of Laboratory Animals [26].

### Anesthesia and animal preparation

Sheep were fasted for 16 to 18 h before the day of operation. During this period, they had unlimited access to water. The animals were pre-medicated with 3 mg/kg body weight (BW) ketamine hydrochloride (Ketasol®100, Dr. E. Graeub AG, Bern, Switzerland) and 0.3 mg/kg BW midazolam (Dormicum® Roche Pharma, Reinach, Switzerland) intravenously for anesthesia induction. Prophylactically, each sheep received a subcutaneous injection of 3 mL tetanus serum (Tetanus-Serum Intervet, ad us. Vet., MSD Animal Health GmbH, Lucerne, Switzerland). Intravenous administration of sodium penicillin (30’000 IU/kg BW) (Penicillin Natrium Streuli® ad us. Vet., Streuli Tiergesundheit AG, Uznach, Switzerland) and gentamycin (4 mg/kg BW) (Vetagent® ad us. Vet., MSD Animal Health GmbH, Lucerne, Switzerland) was used for peri-operative antibiotic treatment. Before orotracheal intubation, the animal was placed in a sternal position and anesthesia was deepened by intravenously administering 2-5 mg/kg BW of propofol (Propofol-®Lipuro 1%, B.Braun Medical AG, Sempach, Switzerland). Anesthesia was maintained throughout the procedure by inhaling 1–2% isoflurane (AttaneTM Isoflurane ad.us.vet., Piramal Enterpr. India, Lyssach, Switzerland) and balanced with a constant rate infusion of propofol (Propofol-®Lipuro 1%, B.Braun Medical AG, Sempach, Switzerland). A constant rate infusion of sufentanil (Sufenta®forte, Janssen-Cilag AG, Zug, Switzerland; 2.5 g/kg BW/h, i.v.) was used to provide intraoperative analgesia, as is standard practice for general anesthesia. Before each surgical incision, deposits of 5–10 mL of the local anesthetic ropivacaine (Ropivacain Sintetica 1%, Sintetica S.A., Mendrisio, Switzerland) were injected subcutaneously into the respective locations. Post-operatively antibiotic treatment and multimodal analgesia was continued for 5 days.

**Fig. 1:**
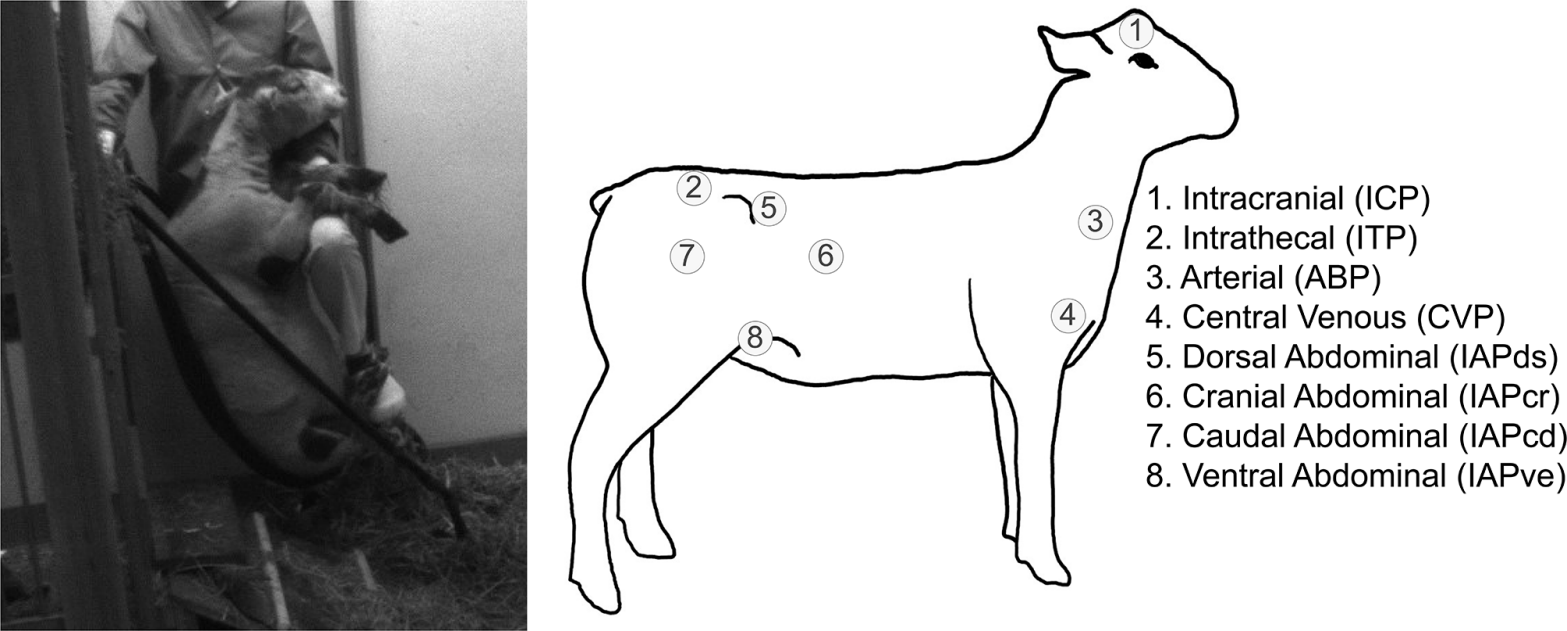
Pressure locations considered in this study in addition to a representative image of a sheep in the chair. 1, intracranial pressure (ICP); 2, intrathecal pressure (ITP); 3, arterial blood pressure (ABP); 4, central venous pressure (CVP); 5, dorsal intra-abdominal pressure (IAPds); 6, cranial intra-abdominal pressure (IAPcr); 7, caudal intra-abdominal pressure (IAPcd); 8, ventral intra-abdominal pressure (IAPve)

### Pressure sensor implantation

All radio-telemetric sensors (Stellar implantable transmitters. TSE Systems GmbH, Bad Homburg, Germany) were surgically implanted in the respective anatomical regions as depicted in Fig. 1. Intravascular telemetric sensors were implanted in the right carotid artery and the right common jugular vein to assess arterial blood pressure (ABP) and central venous pressure (CVP), respectively. A skin incision was made in the mid-cervical region, and the carotid artery and the common jugular vein were dissected and isolated to place the measurement tip approximately 10 cm into the respective vessels. The transmitter device was inserted into a 6 × 4 cm subcutaneous pouch made in the lateral upper neck region. For the implantation of the electrocardiogram leads, an incision of 1–2 cm was made bilaterally on the thorax, near the first rib bone. The insulating cable with a conductive wire at the tip was tunneled from the transmitter body, to both thoracal sites.

A sagittal scalp incision was made for the implantation of the intracranial pressure (ICP) sensor. The pressure sensor was introduced into the right lateral ventricle via a small frontal burr hole trephination – making the right lateral ventricle the cranial reference point for all hydrostatic calculations. To ensure appropriate attachment and avoid CSF leakage, a bone wax plug (Ethicon® Bone wax, Johnson & Johnson Medical Ltd., Livingston, UK) was molded around the existing probe to plug the burr hole in the cranium.

Before the implantation of intrathecal telemetric transmitters, an X-ray of the lumbar spine was taken (Allura Xper FD 20, Philips Medical Systems Netherlands BV, Best, The Netherlands), and the interlaminar window between L6 and L7 was marked. A small laminotomy was performed to implant an intrathecal telemetric sensor in the lumbar region. Subsequently, the catheter measurement tip was advanced cranially within the dural space, thus assuring fluid contact for intrathecal pressure (ITP) measurement. A hemostatic gauze strip (Tabotamp Ethicon, Johnson & Johnson Medical, Neuchatel, Switzerland) was placed on the dura aperture to prevent leakage of CSF from the intrathecal compartment. The distance between ICP and ITP sensor locations measured 64.2 ± 4.8 cm, compared to the equivalent analog human length of 64.1 cm for the same reference points (acquired by the average distance between waist and eye height, which is close to the height of external auditory canal) [27]. To seal the bony defect, ensure adequate catheter fixation and avoid CSF leakage, bone wax additionally was molded around the exiting catheter. A skin incision was then made bilaterally on the flanks of the sheep to implant the intra-abdominal transmitters. The peritoneum was gently opened after the subcutis, and muscular layers were dissected. Two telemetric pressure probes were introduced into each orifice and placed in each of the four quadrants of the abdominal cavity. Subcutaneous pouches were constructed paramedian to the incision sites for the transmitter unit insertion. All sensors measured directly at the tip, i.e. at the source of the pressure.

### Experimental protocol

**Fig. 2:**
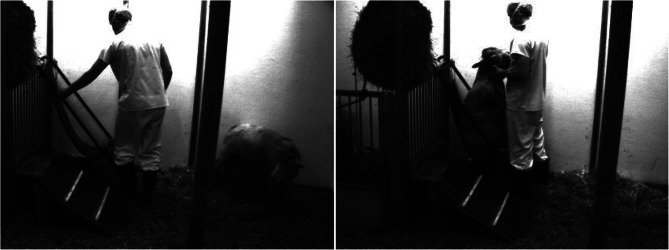
The sheep standing normally before being maneuvered into the chair (left) and held by the trainer for two minutes (right)

The experimental protocol considered in this study was designed to illuminate how the dynamics between various physiologic pressures (Fig. 1) change over the course of a complete standing to upright posture in sheep (Fig. 2). Sheep were allowed to roam freely before being maneuvered into a sheep chair where they were held for two minutes. Then, the sheep were lowered out of the chair and allowed to continue roaming freely. This maneuver was performed repetitively to attain a minimum of 25 maneuvers per animal (N = 6 × 25) with four unique intervals extracted from the continuous measurement: P0, a 30 s phase before the sheep enters the chair to serve as the pre-chair baseline; P1, a 30 s phase right as the sheep enters the chair to show transient changes in pressure; P2, a 30 s phase halftime in the chair approximately between 50 and 80 s; and P3; the final 30 s phase right before the sheep exits the chair.

### Data acquisition and analysis

Telemetric pressure data were acquired in each specific compartment via the Stellar Implantable Transmitters. Each implant included solid-state pressure sensor tipped catheters with an effective measurement range of − 25 to 300 mmHg with a resolution of 0.01 mmHg at 15 to 45 °C (with a resolution of 0.05 °C) and a sampling rate of 100 Hz. In addition, infrared light sensors were included on every implant to allow for data synchronization offline in case of de-synchrony due to each implant having different internal clocks. For ICP and ITP, Type PTA-L2 single-channel glass-tipped implants were implanted with the measurement catheters extending into the CSF spaces to ensure direct fluid pressure measurements. For the measurement of ABP and CVP, dual-channel with onboard ECG leads Type PPBTA-XL titanium-tipped implants were used. Finally, for abdominal measurements, initially, a quad-channel Type PPPPTA-XXL titanium-tipped implant was used, however, this was replaced with two dual-channel Type PPTA-XL titanium-tipped due to the rather larger size of the sensor body. All sensors were wirelessly connected to a workstation running Windows 10 with the acquisition software NOTOCORD-hem (Instem, Stone, Staffordshire, UK). This connection was made via eight D-430,001-REC-04 receivers connected via Cat8 ethernet cables to the workstation.

All data were analyzed using custom scripts written in Python 3.7.10 (Open Source, Python Software Foundation, Willmington, Delaware, United States). Mean pressures and pulse pressures were calculated and are presented. Values are reported as mean ± SD. For all sheep, 20 approximately 120-second-long chair movements were analyzed. Five chairs were excluded per sheep due to mass signal dropout. For each chair, four unique phases were acquired: one baseline before the chair and three values in the chair to acquire a full picture of the pressure dynamics that exist over the duration of the chair. In detail, a 30 s phase before the sheep is moved into the chair (P0), followed by a 30 s phase right after the sheep enters the chair (P1), a 30 s phase around 50-80s (P2), and a final 30 s phase around 90–120 s (P3) were used for the analysis. Within each phase, the mean is taken to acquire a single representative value of that phase. Then, delta of mean pressure measurements $P1-P0, P2-P0, P3-P0$ are used to describe the pressure changes relative to the baseline across the entire duration of the chair. Then, to acquire values representative of all 20 chair movements, the 20 individual phases are averaged within each sheep. Then, results from all sheep are averaged together for comprehensive pressure results representative of our entire cohort (N = 6 Sheep).

Statistical analysis between the differences $P1-P0, P2-P0, P3-P0$ were calculated by repeated measures ANOVA for each pressure, per sheep. Group results are reported in this paper and individual results are reported in the Supplement. Statistical significance was deemed achieved with *p* < .05. To confirm normality, skewness was calculated for each pressure with a value of < 1.96 being deemed acceptable for normality [28]. All results proved to be normally distributed.

### Mean pressure reactions

**Fig. 3:**
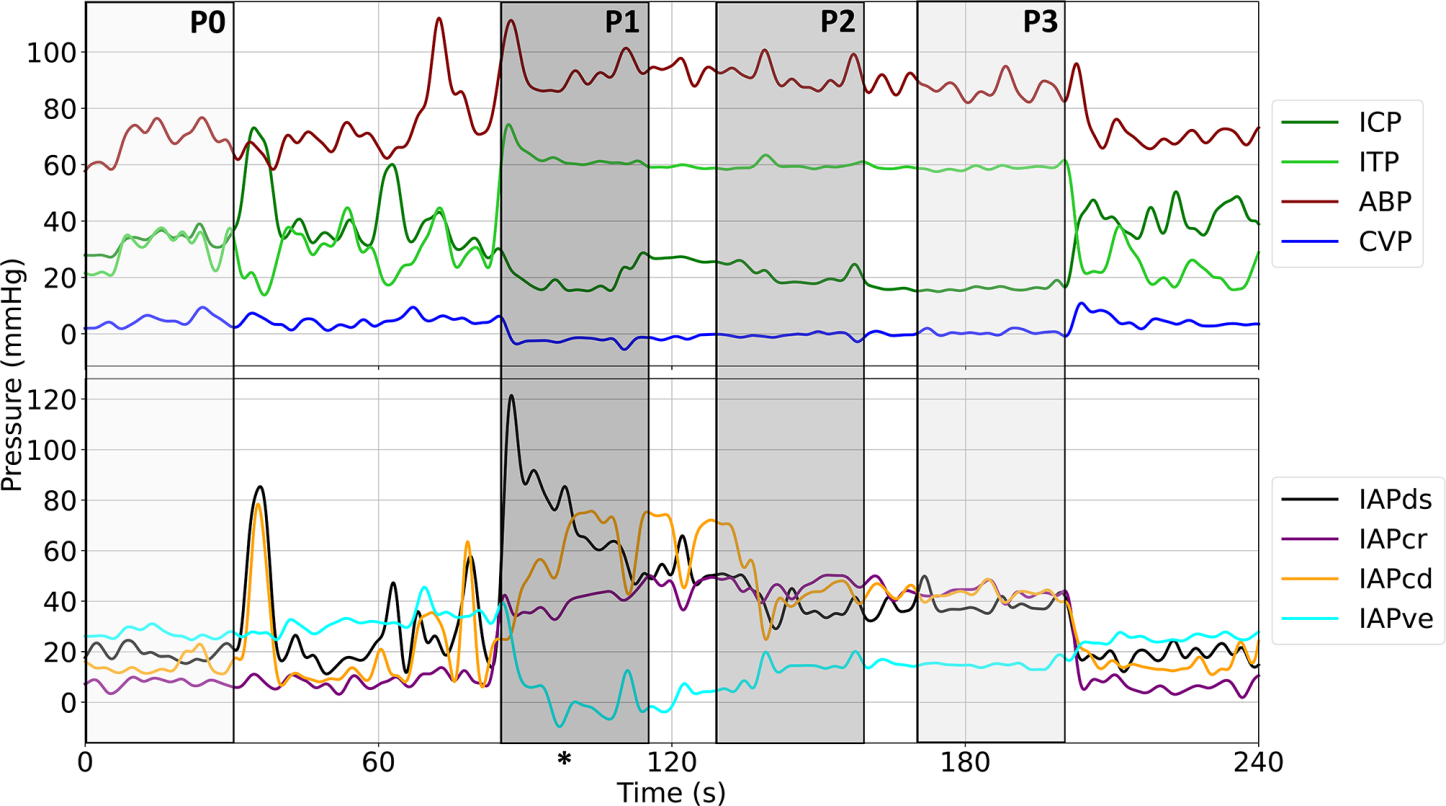
Exemplary recording of one transfer into and out of the chair to visualize the different phases considered in pressure change calculations. The lightest grey region denotes P0, the darkest grey P1, middle grey P2, and light grey P3, respectively. The asterisk (*) marks the time of the close-up view depicted in Fig. 4. ICP, intracranial pressure; ITP, intrathecal pressure; ABP, arterial blood pressure; CVP, central venous pressure; IAPds, dorsal intraabdominal pressure; IAPcr, cranial intraabdominal pressure; IAPcd, caudal intraabdominal pressure; IAPve, ventral intraabdominal pressure

The calculation of mean pressure changes was performed to acquire insights into the hydrostatic effects on each compartmental pressure. All data were preprocessed prior to analysis. Outliers were rejected by using a z-score rejection method with a σ_crit_ of 3. Then, to remove cardiac and respiratory effects, the data were lowpass filtered using a 4th order forward/backward Butterworth filter with a cutoff frequency of 0.1 Hz (10 s periods) [22]. The differences between chair phases and baseline were calculated as the numerical difference between $P1-P0, P2-P0, P3-P0$, respectively (Fig. 3).

**Pulse pressure reactions**.

**Fig. 4:**
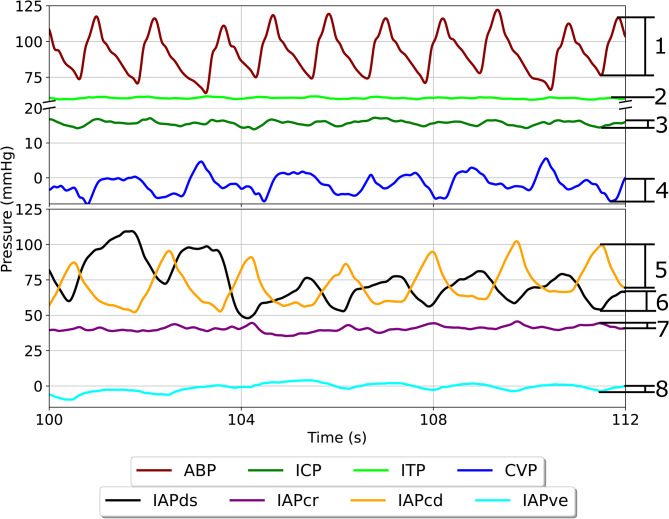
Close-up view of the same sheep as in Fig. 3 exact timing denoted with an asterisk. The difference in peaks and troughs are used to calculate the pulse pressure for each pressure considered. 1, ABP_amp_, arterial blood pressure amplitude; 2, ITP_amp_, intrathecal pressure amplitude; 3, ICP_amp_, intracranial pressure amplitude; 4, CVP_amp_, central venous pressure amplitude; 5, IAPcd_amp_, caudal intraabdominal pressure 6, IAPds_amp_ dorsal intraabdominal pressure amplitude; 7, IAPcr_amp_, cranial intraabdominal pressure amplitude; 8, IAPve_amp_, ventral intraabdominal pressure amplitude

Reactions in pulse pressure are calculated as the difference between peaks and troughs of each pressure source’s dominant frequency (Fig. 4). For ICP, ITP, ABP, and CVP, the dominant frequency is the cardiac cycle, therefore systolic and diastolic peaks are used to calculate pulse pressure. For abdominal pressure, the dominant frequency is the respiratory cycle, therefore peaks and troughs in the respiratory waveform are used to calculate pulse pressure. Pulse pressures for each individual waveform are calculated and averaged for each phase P0, P1, P2, and P3, as described above. Then, differences are calculated as $P1-P0, P2-P0, P3-P0$ to acquire changes in pulse pressure.

## Results

### Pressure reactions

**Fig. 5:**
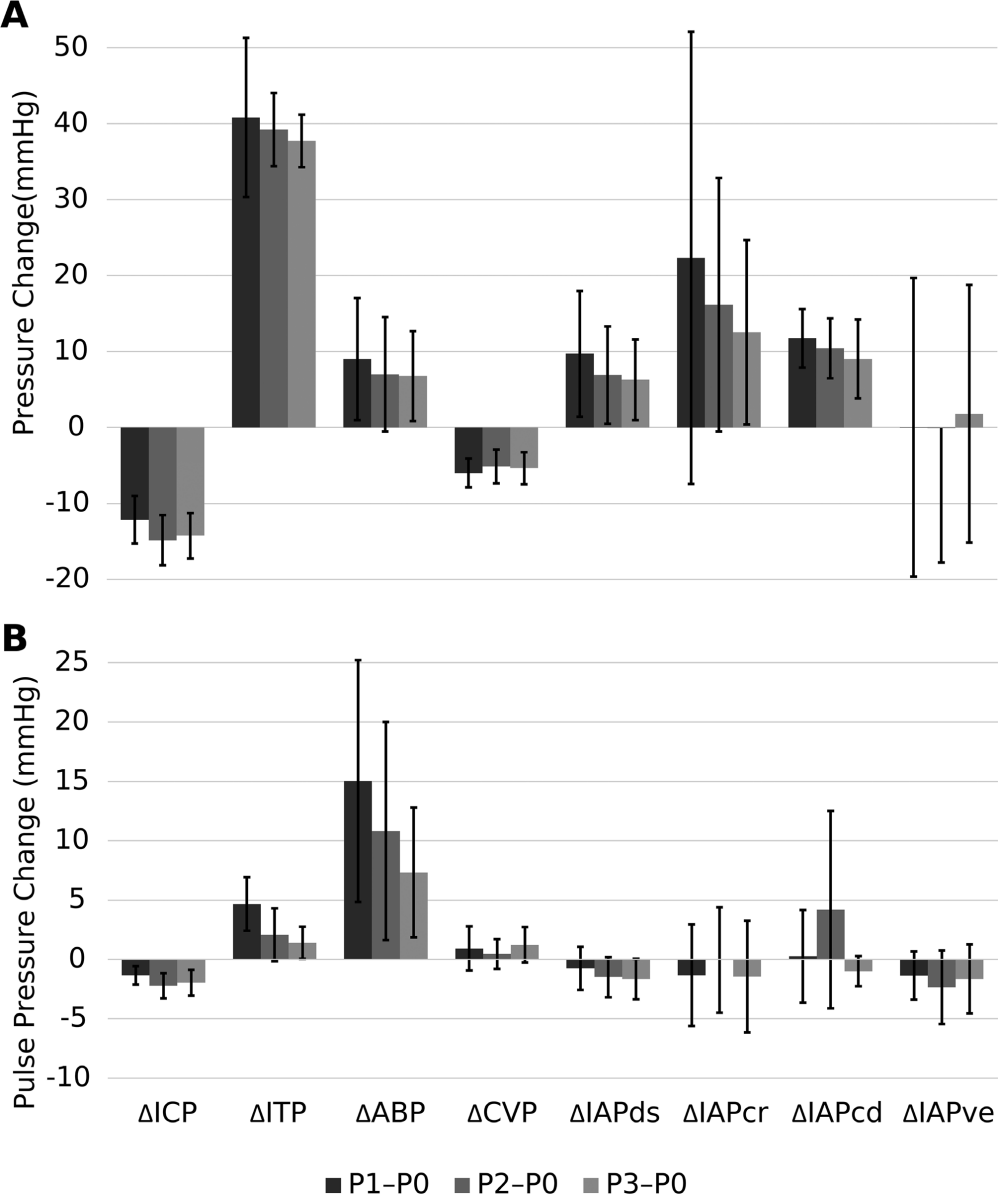
**A**: Mean pressure changes and respective standard deviations (SD) across all sheep in all compartments from P1, P2, and P3 to P0 and **B**: change in pulse pressure from P1, P2, and P3 to P0. Darkest bars represent the difference between P1 and P0; middle bars the difference between P2 and P0, and the lightest bars the difference between P3 and P0, respectively, including one standard deviation. For individual sheep-specific results, please see Fig. 1S; Tables 1S and 2S in the Supplement. ICP, intracranial pressure; ITP, intrathecal pressure; ABP, arterial blood pressure; CVP, central venous pressure; IAPds, dorsal intra-abdominal pressure; IAPcr, cranial intra-abdominal pressure; IAPcd, caudal intra-abdominal pressure; IAPve, ventral intra-abdominal pressure

**Table 1:** Numerical results of the differences between P1 and P0, P2 and P0, and P3 and P0 for all pressures considered in **top** mean pressure and **bottom** pulse pressure including one standard deviation. Statistical significance is shown for each pressure with significance determined by p-value < 0.05. Non-significant values are marked with NS. ICP, intracranial pressure; ITP, intrathecal pressure; ABP, arterial blood pressure; CVP, central venous pressure; IAPds, dorsal intra-abdominal pressure; IAPcr, cranial intra-abdominal pressure; IAPcd, caudal intra-abdominal pressure; IAPve, ventral intra-abdominal pressure

Mean Pressure	ΔICP (mmHg)	ΔITP (mmHg)	ΔABP (mmHg)	ΔCVP (mmHg)	ΔIAPds (mmHg)	ΔIAPcr (mmHg)	ΔIAPcd (mmHg)	ΔIAPve (mmHg)
**P1 – P0**	−12.1 ± 3.1	40.8 ± 10.5	9.0 ± 8.0	−5.9 ± 1.9	9.7 ± 8.3	22.3 ± 29.8	11.7 ± 3.9	0.03 ± 19.6
**P2 – P0**	−14.8 ± 3.3	39.2 ± 4.8	7.0 ± 7.5	−5.1 ± 2.2	6.9 ± 6.4	16.1 ± 6.4	10.4 ± 3.9	−0.1 ± 17.7
**P3 – P0**	−14.2 ± 3.0	37.7 ± 3.5	6.7 ± 5.9	−5.3 ± 2.1	6.2 ± 5.3	12.5 ± 12.1	9.0 ± 5.2	1.8 ± 17.0
**p-value**	**< 0.001**	**0.019**	NS	NS	**0.0116**	**0.042**	**0.014**	NS
**Pulse Pressure**	Δ**ICP**_**amp**_**(mmHg)**	Δ**ITP**_**amp**_**(mmHg)**	Δ**ABP**_**amp**_**(mmHg)**	Δ**CVP**_**amp**_**(mmHg)**	Δ**IAPds**_**amp**_**(mmHg)**	Δ**IAPcr**_**amp**_**(mmHg)**	Δ**IAPcd**_**amp**_**(mmHg)**	Δ**IAPve**_**amp**_**(mmHg)**
**P1 – P0**	−1.3 ± 0.7	4.7 ± 2.3	15.0 ± 10.2	0.9 ± 1.9	−0.7 ± 1.8	−1.3 ± 4.2	0.3 ± 3.9	−1.4 ± 2.0
**P2 – P0**	−2.2 ± 1.0	2.1 ± 2.2	10.8 ± 9.2	0.5 ± 1.2	−1.5 ± 1.7	0.0 ± 4.4	4.2 ± 8.3	−2.3 ± 3.1
**P3 – P0**	−2.0 ± 1.1	1.4 ± 1.4	7.3 ± 5.5	1.2 ± 1.5	−1.7 ± 1.7	−1.4 ± 4.7	−1.0 ± 1.3	−1.6 ± 2.9
**p-value**	**< 0.001**	**< 0.001**	**< 0.001**	NS	**< 0.001**	**0.006**	**< 0.001**	NS

Figure 5; Table 1 show results over all sheep in all mean pressure and pulse pressures measured for the differences between chair phases P1, P2, P3, and P0. ICP decreased both in mean pressure and pulse pressure when in the upright posture while ITP increased in both. ABP increased in mean pressure while CVP decreased, with both increasing in pulse pressure. There were varied extents of changes in the abdominal pressures, with IAPcr and IAPcd increasing in mean with the upright posture while IAPds decreased. IAPve reacted considerably different across animals, as shown by the large SD. There were more varied results in abdominal pulse pressures, with no discernable patterns emerging. For full results showing intra-sheep variations, see supplementary Fig. 1S and Tables 1S and 2S.

## Discussion

### Mean pressure reactions

During the in-the-chair maneuver, there are very distinct significant behaviors in the mean pressure reactions of ICP and ITP: firstly, they are opposite yet unequal – ICP experienced changes of (P1, P2, P3 compared to P0) − 12.1, − 14.8, and − 14.2 mmHg while ITP experienced changes of + 40.8, + 39.2, and + 37.7 mmHg across the same timeline. In cats, it was found that there exist two unique hydrostatic columns acting over the craniospinal space: the ventricles to Foramen Magnum (FM) (cranial column) and the FM to lumbar dural space (spinal column) [29]. If this were to be true, in our sheep, the changes as dictated by the hydrostatic column in the upright position are approximately 2.0 mmHg on the cranial column, given the few cm of fluid above the measurement point and approximately 42.4 mmHg on the spinal column, relative to the vertical distances between the ventricles and the cranium (approximately 2.0 cm) and the FM and lumbar space measurement point (approximately 57.3 cm) in the upright posture. In our sheep, ICP experienced a change of approximately − 12.1 (P0 to P1) and − 14.8 (P0 to P3) mmHg, suggesting a calculated hydrostatic column of around 19 cm if one were to assume that the entirety of the change occurs due to hydrostatics, quite larger than the anatomical reality of there only being a few cm of fluid above the intraventricular measurement location. This supports the idea that ICP dynamics are dictated more heavily by alternative sources, such as changes in cerebral blood pressure and volume. The change in ITP agrees with what the spinal hydrostatic column would expect, near the FM, suggesting that the hydrostatic column influences lumbar CSF pressures to a greater extent than some compensatory mechanism. Interestingly, when the full craniospinal pressure change, around 70 mmHg is considered, a full hydrostatic column of around 70 cm is calculated, which is larger than the distance between the ICP and ITP measurement locations. This further argues that there is indeed more than just hydrostatics that dictate pressure changes in CSF pressures, and that there are other influences at play. Secondly, ICP experiences a sudden drop in pressure in response to the upright posture (P1), then decreases further albeit at a much slower rate (P2), and slightly increases near the end of the chair (P3). The change at P1 may be attributed to a large decrease in intracranial venous pressure which causes the stark drop in ICP, as supported by reductions in CVP being previously reported as influencers of ICP decreases in mice [30]. ICP does indeed continue to drop in P2, albeit much slower than what occurred at P1. This may be indicative of a venous collapse, thus reducing the venous outflow, and attempting to compensate and stabilize ICP. This effect has been previously reported by our group in an acute, anesthetized ovine setting [23]. Then, in P3, it is observed that ICP begins to slowly increase and ITP slowly decrease, which may be further evidence of the effectiveness of this venous compensation. This compensatory mechanism will effectively cause CSF formation to outpace absorption and outflow, thus causing a slow increase of ICP (formation-heavy) and slow decrease of ITP (absorption-heavy). Furthermore, this may be paired with a collapse of the spinal compartment, as was reported in cats [29]. ITP, on the other hand, experiences a peak pressure right after entering the upright posture and continues to decrease over the duration of the chair. It is possible that the sudden change in posture not only causes the hydrostatic column acting on ITP to change, but also potentially an increase in the cranial-caudal flow of CSF. The sudden change may cause a rush of CSF out of the cranium and into the lumbar CSF space, and therefore an acute rise in ITP and acute reduction in ICP. However, it has been previously reported that there is a collapse of the spinal CSF compartment in the upright posture in cats, therefore it is possible that this, paired with the already reported venous compensation in sheep, combine to limit the amount of CSF transfer caudally and cause resorption to outpace formation in the spinal compartment [29].

Blood pressure revealed similar patterns to the posture change to ITP – with non-significant changes of 9.0, 7.0, and 6.7 mmHg when compared to the initial pre-chair baseline phase. This can be attributed to natural stress responses to the non-physiologic upright posture. There have been studies in humans that reported ABP as decreasing from supine to standing postures, which challenge these results [31]. However, as sheep are quadrupeds, they are not physiologically adapted to being in the upright posture for extended periods of time, potentially leading to increased stress levels when compared to their standing baseline. However, it is important to note the decreasing pressure trend of the ABP as the sheep calms down during the chair and effectively becomes acclimatized to the position. CVP experienced drops in pressure, showing changes of − 6.0, − 5.1, and − 5.3 mmHg when compared to baseline. In humans, an upright posture causes a redistribution of venous volume to the distal veins, decreases CVP via gravitational forces acting on the highly compliant venous structure and decreases in venous return via thoracic venous blood volumes [32]. This, paired with a potential drop in thoracic pressure can yield this seemingly large drop in CVP.

Abdominal pressures show clear dependence on the posture change. Responses varied quite considerably in each sheep, as shown in the large standard deviation in all but IAPcd and the individual results shown in the supplementary material. Nonetheless, IAPds, IAPcr, and IAPcd showed similar trends to ITP and ABP, albeit for different reasons with IAPve lacking significance. The ovine abdomen is comprised of organs, fluid, and gas in seemingly heterogeneous organization. It is possible that the sudden change in posture caused a buildup of pressure in the cranial and caudal locations that dissipated over the duration of the chair due to rearrangement of abdominal content into a more optimal layout due to the continued influence of gravity. In a clinical study of humans following liver transplant, IAP was measured at the upper and lower abdominal walls following the surgery and then exposed to varying levels of tilt. It was reported that a clinically significant difference in upper and lower IAP exists following intermittent posture change [33]. In this clinical study, a percent difference of 18% was reported, different than what is observed in the cranial and caudal abdominal pressures of our sheep. Nevertheless, the different responses further support that the abdominal cavity should not be considered as a unified entity, rather a set of different related sub-compartments.

### Pulse pressure reactions

Physiologic pulse pressure is a function of natural compliance and volume changes. Whenever a confined fluid cavity (e.g., the intrathecal sac) experiences a volumetric increase, a reduction in compliance occurs due to the cavity “stretching” and becoming stiffer. This effectively reduces the cavity’s ability to attenuate the fluids pulsations, and therefore leads to the pulse pressure of that cavity to increase (i.e., as mean pressure increases, pulse pressure increases). The contrary is also true, a loss of volume tends to lead to a decrease in pulse pressure (i.e., as mean pressure decreases, pulse pressure decreases). In the CSF system, compliance also includes that natural “stiffness” of the tissue and the surrounding blood pressures that push against the tissue expansion. A retrospective study of 25 patients with probable normal pressure hydrocephalus (NPH) was conducted to evaluate the relationship between mean pressure and pulse pressure in the CSF space. This study reported three distinct phases to describe the relationships between mean pressure and pulse pressure: a constant phase at lower pressures (i.e. changes in mean pressure have no impact on pulse pressure), a transitional phase at mid-pressures, and a linear phase at higher pressures (i.e. changes in mean pressure have a linear impact on pulse pressures) [34]. In our ovine study, the changes in mean ICP_amp_ and ITP_amp_ as a result of the posture change yielded changes in pulse pressures of − 1.3, − 2.2, and − 2.0 mmHg in ICP and + 4.7, + 2.1, and + 1.4 mmHg in ITP. However, before the sheep entered the chair, ICP_amp_ and ITP_amp_ were comparable at 4.1 ± 1.0 mmHg and 3.8 ± 1.1 mmHg, respectively, leading ITP_amp_ to be considerably larger in absolute terms than ICP_amp_ in the upright posture (approximately 2 − 3 mmHg for ICP_amp_ and 6 − 8 mmHg for ITP_amp_). Assuming that there is an inverse relationship with pressure and compliance, it is possible to see this as evidence that, in the upright posture in sheep, it is actually the cranial compartment that provides that majority of the cranio-spinal compliance, which agrees with the work of Gehlen et al. reporting that only 10% of cranio-spinal compliance is provided by the spinal compartment in the upright posture in humans [35]. It has also been previously reported that there is a redistribution of venous blood volume out of the brain in the upright posture, which may also play a role in increasing ventricular compliance, and therefore decreasing ICP_amp_ [36]. These results also support the cranial-caudal rush of CSF to the lumbar CSF space, further reducing compliance in the lumbar space and causing the large increase in ITP_amp_. In essence, there is a cardiac-cycle induced pressure pulse in ICP which then propagates caudally towards the thecal space, and is amplified as the wave interacts with dural walls with continually reduced compliance. Thus, causing the larger ITP_amp_ when compared to ICP_amp_. There is one notable study in shunted hydrocephalic elderly adults from Farahmand et al. [37]. noting a slight increase in ICP_amp_ as a result of moving from supine to sitting to standing posture, which suggests that compliance was either unchanged or marginally reduced by the change in posture and that the redistribution of CSF is not the primary influencer of ICP_amp_ dynamic changes. Pulse pressure is a combination of natural compliance and volume change that drives the pulsatility (i.e. blood volume change in the case of CSF). In this ovine study, a change of around − 5 mmHg in CVP is observed, which supports the idea of a redistribution of venous blood towards the legs in the upright posture. It is possible that, in sheep, the effect of the cerebral venous system on intracranial compliance (ICC) is greater than that of humans, which may have caused the seemingly contrasting results. Moreover, trends in ICP_amp_ and ITP_amp_ followed those of mean pressure, further supporting the combination of CSF and blood redistribution to be influencer of cranio-spinal pulse dynamics.

ABP_amp_ also followed its mean pressure trend, experiencing changes of 15.0, 10.8, and 7.3 mmHg from P1, P2, and P3 compared to baseline. This supports the idea of stress being a key player in ABP modulation during upright posture change in sheep, and that the sheep’s’ stress levels reduce during the duration of the chair, as evidence by the reduction in pulse pressure over time. In humans, stress has been shown to influence increases in mean ABP via stimulation of the sympathetic nervous system to produce large amounts of vasoactive agents that contribute to increased blood pressure. Arteries, especially large-lumen arteries, are lowly compliant due to the requirement that they transmit cardiac pulses over long distances, are firmer than veins, and constrictions may cause the vessel walls to stiffen even more [38]. The paired action of the increased blood pressure and vasoconstricting hormones may cause a synergistic reaction to increase ABP_amp_ by a larger relative percentage than the increase in mean pressure would suggest. Even though actions were taken to reduce sheep stress; sheep were acclimatized to the experimental environment and the individual trainers before any data collection was acquired, the chair movement itself is not physiologic, which may have led to increased stress hormone release. CVP, being an indicator of venous return and right atrial pressure, usually remains relatively stable due to the measurement locations proximity to the heart. Therefore, the limited response of CVP_amp_ suggests that this physiologic control pathway remained intact during the duration of the chair – even with the results lacking significance.

Abdominal pressures, while responding with large changes in mean pressure, have markedly lower responses in pulse pressure. As the abdominal compartment is a mix of solid and hollow organs filled with fluid and gases, the distribution of pressure may have a lower impact on pulse pressure responses in the abdominal space than those of the CSF or arteriovenous compartments. This appears to be subject specific as responses varied considerably from sheep to sheep, causing large standard deviations. Abdominal responses, much like the makeup of abdominal compartments, appears to be subject-specific, and the varied responses further support the idea that the abdomen is comprised of multiple different heterogeneous components and perhaps should not be considered as a unified homogeneous entity in clinical practice.

### Limitations

One can only speculate the effects of stress responses on changes in ABP observed in this study. Stress hormones, particularly responses to repetitive “un-natural” positions, may be a limitation in the ABP results. To confirm these findings, comparative measurements of stress hormones during these movements and heart rate data are currently being investigated to further our understanding of the relationship between stress and pressure. Moreover, quadrupeds are physiologically adapted to a CSF axis that is commonly horizontal compared to humans where the CSF axis is firstly vertical most of the time, and secondly may experience larger changes in CSF volume. This may also lead to limitations in translating CSF-specific relationships to humans, however similar physiological adaptations have been previously observed between sheep and humans. Drift was observed in all sensors such that absolute pressures (namely baseline pressures) were limited to be validly calculated and reported within the context of this study. While changes in pressure can still be correctly analyzed, absolute pressures would provide more information regarding hydrostatic differences between pressure sources. The authors continue to work on numerically remedying this drift for future studies. Finally, there were dropouts in the sensor data occasionally during the tilt movement, causing some of the data in each sheep to be excluded from calculations. While most of the data was able to be captured and calculated, it is possible that some quantitative results may be slightly different if all data could have been represented.

## Conclusions

In a chronic awake ovine in-vivo trial, eight pressures have been measured simultaneously. All pressures showed posture-dependent changes. Reactions in mean ITP agree with the calculated hydrostatic column between the FM and thecal measurement location, indicating hydrostatic influences as a key influencer of mean pressure change within the lumbar CSF space. The differing responses of ICP and ITP in mean pressure motivates that there does indeed exist a level of physiologic compensation to limit ICP change with respect to ITP. ICP results support hydrostatics to play a minimal role in pressure dynamics, with changes in intracranial volume playing a larger role. ITP_amp_ being considerably larger than ICP_amp_ in the upright posture agrees with previously reported literature that, in the upright posture, the cranial compartment provides the majority of craniospinal compliance. Large dynamic changes in IAP vary across abdominal locations, motivating the abdominal cavity not to be considered as a unified entity, rather a set of sub-compartments with unique dynamics.

### Electronic supplementary material

Below is the link to the electronic supplementary material.

Supplementary Material 1
