# Supplementary material for: The influence of upright posture on craniospinal, arteriovenous, and abdominal pressures in a chronic ovine in-vivo trial
Source: Fluids Barriers CNS. 2023 Nov 9;20:83. doi: 10.1186/s12987-023-00485-6 (PMC10634040; doi:10.1186/s12987-023-00485-6)
Supplement: Supplementary file 1 — Supplementary Material 1 [file 12987_2023_485_MOESM1_ESM.docx]

Supplement to:

The influence of upright posture on craniospinal, arteriovenous, and abdominal pressures in a chronic ovine in-vivo trial

Anthony Podgoršak^1^, Nina Eva Trimmel^2^, Markus Florian Oertel^3^, Margarete Arras^2^, Miriam Weisskopf^2^, Marianne Schmid Daners^1^

^1^ Department of Mechanical and Process Engineering, ETH Zurich, Zurich, Switzerland

^2^ Center for Preclinical Development, University Hospital Zurich, University of Zurich, Zurich, Switzerland

^3^ Department of Neurosurgery, University Hospital Zurich, University of Zurich, Zurich, Switzerland

**Corresponding Author:** Dr. Marianne Schmid Daners ([marischm@ethz.ch](mailto:marischm@ethz.ch))

https://doi.org/10.1186/s12987-023-00485-6


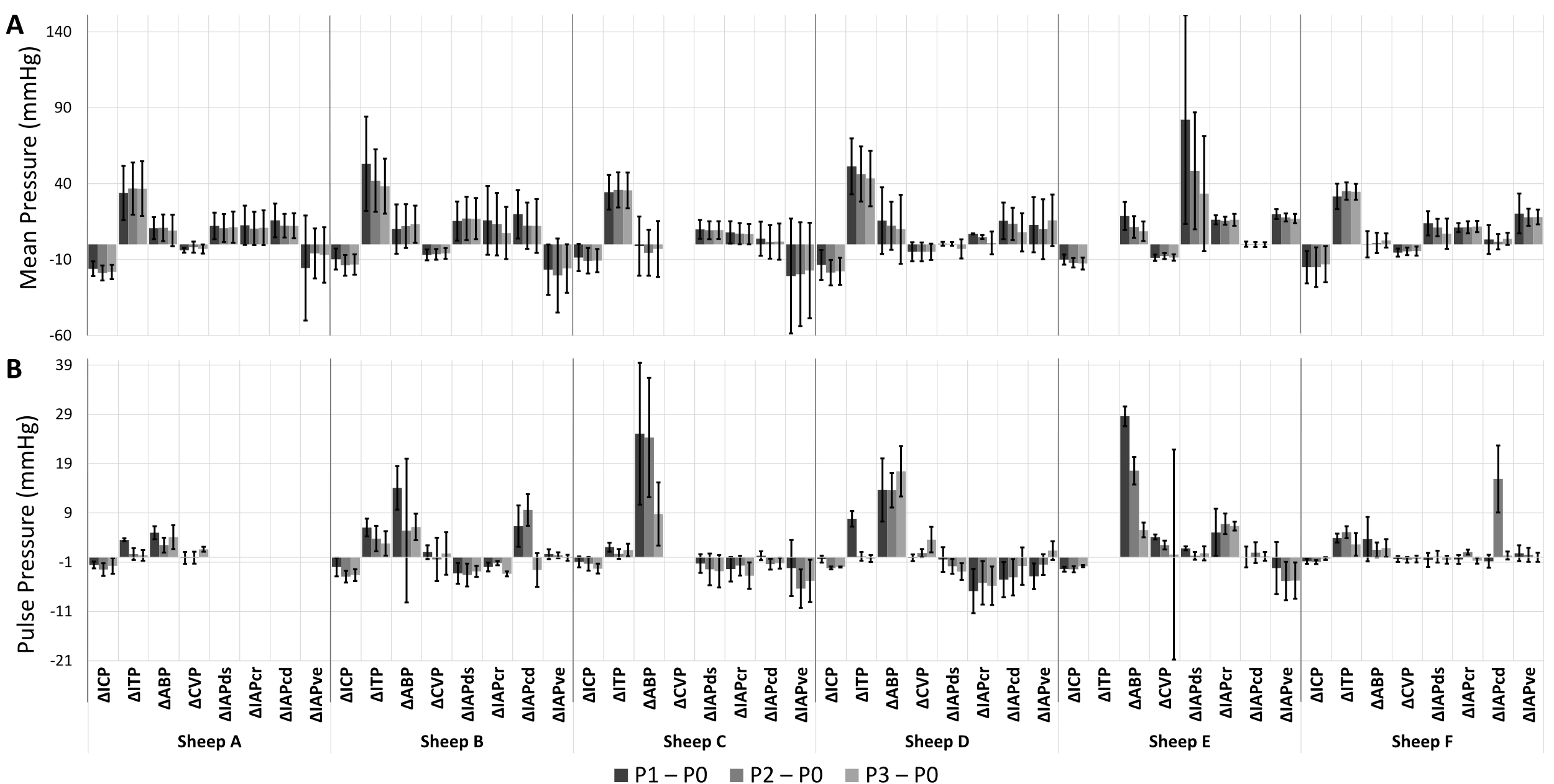


Figure 1S **A** Changes in mean pressure and **B** changes in pulse pressure from the phases P1–P0 (Dark Grey), P2–P0 (Middle Grey), and P3–P0 (Light Grey) for all sheep in mmHg±SD. Error bars indicate SD. Δ, Change in; ICP, intracranial pressure; ITP, intrathecal pressure; ABP, arterial blood pressure; CVP, central venous pressure; IAPds, dorsal intra-abdominal pressure; IAPcr, cranial intra-abdominal pressure; IAPca, caudal intra-abdominal pressure; IAPve, ventral intra-abdominal pressure.

Figure 1S, Table 1S and Table 2S show the sheep-specific results for the differences P1–P0, P2–P0, and P3–P0. Significance varied considerably within each sheep, as opposed to viewing the data on the cohort scale, where more significance was derived. ITP carried consistently strong positive reactions to the upright posture, whereas ICP consistently reacted by decreasing. A similar relationship was observed in the pulse pressures, where the reactions are inverted, albeit stronger in the lumbar compartment. Abdominal mean and pulse pressures varied strongly between sheep, even within the same pressure source. Within Table 1S, there were periods of dropouts in the central venous pressure (CVP) of Sheep C and intrathecal pressure (ITP) of Sheep E. In consultation with the sensor manufacturer, the authors believe this to be attributed to breaking of the glass sensor tip, which caused the internal wiring to become saturated with blood and resulted in a short circuit. Within Table 2S, there were more periods where no valid data could be collected, specifically the abdominal pulse pressures of sheep A in addition to CVP of Sheep C and ITP of Sheep E. Similarly in consultation with the manufacturer, the authors believe this lack of abdominal pulse pressures in Sheep A is due to the accumulation of soft tissue on the titanium sensor tips, effectively attenuating any respiratory effects that would have presented themselves on the waveform. Therefore, the abdominal pulse pressure data from Sheep A was unable to be included in the analysis.

Table 1S Comprehensive results for individual sheep mean pressure changes of the differences P1–P0, P2–P0, and P3–P0 in mmHg±SD and significance, as calculated by repeated measures ANOVA. Significance was defined with p<.05. NS, Not Significant. N/A denotes periods where no valid data could be collected; these data points were excluded from analysis. ICP, intracranial pressure; ITP, intrathecal pressure; ABP, arterial blood pressure; CVP, central venous pressure; IAPcr, cranial intra-abdominal pressure; IAPca, caudal intra-abdominal pressure; IAPds, dorsal intra-abdominal pressure; IAPve, ventral intra-abdominal pressure.

| Sheep | Δ | ΔICP (mmHg) | ΔITP  (mmHg) | ΔABP (mmHg) | ΔCVP (mmHg) | ΔIAPcr (mmHg) | ΔIAPca (mmHg) | ΔIAPds (mmHg) | ΔIAPve (mmHg) |
| --- | --- | --- | --- | --- | --- | --- | --- | --- | --- |
| A | P1 – P0 | –16.0±4.8 | 33.8±17.8 | 10.7±7.2 | –3.9±1.5 | 12.2±8.7 | 12.6±12.8 | 15.8±11.1 | –15.6±34.6 |
|  | P2 – P0 | –18.8±4.9 | 36.8±17.1 | 10.9±8.8 | –1.8±3.6 | 10.8±9.2 | 10.5±10.9 | 12.3±7.8 | –5.9±16.5 |
|  | P3 – P0 | –18.2±4.8 | 36.7±18.0 | 9.1±10.5 | –3.0±3.0 | 11.3±10.3 | 11.0±11.4 | 12.2±8.3 | –6.9±18.2 |
|  | p–value | NS | NS | NS | NS | NS | NS | NS | NS |
| b | P1 – P0 | –9.6±6.8 | 53.0±31.0 | 10.1±16.2 | –6.9±3.6 | 15.4±12.8 | 15.8±22.6 | 19.9±15.9 | –16.6±16.5 |
|  | P2 – P0 | –13.7±6.8 | 42.0±20.5 | 12.0±14.4 | –6.5±3.5 | 17.0±14.3 | 13.3±20.5 | 12.3±15.2 | –20.5±14.3 |
|  | P3 – P0 | –13.2±6.7 | 38.3±18.1 | 13.3±12.3 | –5.9±3.5 | 16.9±13.6 | 7.5±18.1 | 12.2±17.7 | –15.9±15.9 |
|  | p–value | <.001 | <.001 | NS | .001 | .04 | .02 | .04 | NS |
| c | P1 – P0 | –8.6±8.9 | 34.3±11.4 | –1.1±19.5 | N/A | 9.8±6.2 | 7.9±7.2 | 3.7±11.2 | –20.8±37.8 |
|  | P2 – P0 | –10.7±8.3 | 35.9±11.6 | –5.5±15.0 | N/A | 9.3±5.8 | 7.0±6.9 | 1.6±10.9 | –19.6±34.4 |
|  | P3 – P0 | –10.6±7.6 | 35.5±11.8 | –3.0±18.4 | N/A | 9.4±5.8 | 6.7±6.6 | 1.8±11.9 | –17.1±31.4 |
|  | p–value | .01 | NS | NS | N/A | NS | .01 | NS | NS |
| D | P1 – P0 | –13.5±9.8 | 51.4±18.4 | 15.7±21.8 | –4.8±6.2 | 0.4±1.2 | 6.9±0.3 | 15.5±12.0 | 12.9±18.1 |
|  | P2 – P0 | –18.5±8.3 | 46.4±18.0 | 12.3±15.8 | –4.9±6.1 | 0.3±1.2 | 4.9±1.3 | 13.5±10.6 | 10.0±19.7 |
|  | P3 – P0 | –17.7±8.8 | 43.4±18.2 | 10.0±22.6 | –4.8±6.3 | –3.0±6.3 | 1.0±7.5 | 8.0±12.6 | 15.9±16.9 |
|  | p–value | <.001 | NS | NS | NS | NS | NS | NS | NS |
| E | P1 – P0 | –10.0±3.3 | N/A | 18.6±9.3 | –8.8±2.1 | 82.1±68.6 | 16.2±2.9 | 0.2±1.7 | 19.9±3.3 |
|  | P2 – P0 | –12.1±3.1 | N/A | 11.5±7.1 | –7.7±1.9 | 48.4±38.4 | 15.6±2.6 | –0.1±1.6 | 17.7±2.6 |
|  | P3 – P0 | –12.6±3.8 | N/A | 8.6±6.4 | –8.6±2.1 | 33.4±37.9 | 16.2±3.9 | –0.1±1.5 | 17.0±3.1 |
|  | p–value | .005 | N/A | <.001 | NS | .01 | NS | NS | .03 |
| F | P1 – P0 | –15.0±10.6 | 31.6±8.3 | 0.1±8.7 | –5.4±2.6 | 13.9±7.9 | 11.1±2.9 | 3.2±9.4 | 20.3±13.1 |
|  | P2 – P0 | –15.0±13.0 | 35.1±5.7 | 0.9±6.7 | –4.6±2.5 | 11.1±5.7 | 11.3±4.0 | 1.8±5.0 | 17.9±5.6 |
|  | P3 – P0 | –13.1±11.9 | 34.7±5.2 | 2.6±4.6 | –4.3±3.0 | 7.1±9.9 | 11.8±3.7 | 3.6±3.9 | 18.0±4.8 |
|  | p–value | NS | NA | NS | NS | NS | NS | NS | NS |

Table 2S Comprehensive results for individual sheep pulse pressure changes of the differences P1–P0, P2–P0, and P3–P0 with standard deviations and significance, as calculated by repeated measures ANOVA. Significance was defined with p<.05. NS, Not Significant.N/A denotes periods where no valid data could be collected; these data points were excluded from the analysis. ICP_amp_, intracranial pressure amplitude; ITP_amp_, intrathecal pressure amplitude; ABP_amp_, arterial blood pressure amplitude; CVP_amp_, central venous pressure amplitude; IAPcr_amp_, cranial intra-abdominal pressure amplitude; IAPca_amp_, caudal intra-abdominal pressure amplitude; IAPds_amp_, dorsal intra-abdominal pressure amplitude; IAPve_amp_, ventral intra-abdominal pressure amplitude.

| Sheep | Δ | ΔICP_amp_ (mmHg) | ΔITP_amp_  (mmHg) | ΔABP_amp_ (mmHg) | ΔCVP_amp_ (mmHg) | ΔIAPcr_amp_ (mmHg) | ΔIAPca_amp_ (mmHg) | ΔIAPds_amp_ (mmHg) | ΔIAPve_amp_ (mmHg) |
| --- | --- | --- | --- | --- | --- | --- | --- | --- | --- |
| A | P1 – P0 | –1.6±0.7 | 3.5±0.3 | 5.0±1.3 | –0.1±1.2 | N/A | N/A | N/A | N/A |
|  | P2 – P0 | –2.5±1.3 | 0.6±1.2 | 2.5±1.5 | –0.1±1.2 | N/A | N/A | N/A | N/A |
|  | P3 – P0 | –1.8±1.6 | 0.4±1.1 | 4.1±2.4 | 1.6±0.5 | N/A | N/A | N/A | N/A |
|  | p–value | NS | <.001 | NS | .01 | N/A | N/A | N/A | N/A |
| b | P1 – P0 | –2.0±1.9 | 6.0±1.8 | 14.1±4.4 | 1.0±1.4 | –2.0±0.9 | 6.3±4.2 | –3.3±2.1 | 0.6±1.0 |
|  | P2 – P0 | –3.9±1.2 | 3.8±2.6 | 5.4±14.6 | –0.4±4.4 | –1.2±0.4 | 9.6±3.2 | –3.6±2.3 | 0.4±0.6 |
|  | P3 – P0 | –3.6±1.2 | 2.8±2.5 | 6.2±2.7 | 0.8±4.3 | –3.4±0.5 | –2.6±3.4 | –2.9±1.1 | –0.1±0.6 |
|  | p–value | <.001 | <.001 | NS | NS | .<.001 | <.001 | NS | NS |
| c | P1 – P0 | –0.9±1.1 | 2.0±0.9 | 25.1±14.4 | N/A | –2.4±2.5 | 0.3±0.9 | –1.3±1.9 | –2.2±5.7 |
|  | P2 – P0 | –1.3±1.4 | 0.7±1.0 | 24.3±12.1 | N/A | –1.7±2.0 | –1.41.1 | –2.5±3.2 | –6.3±3.9 |
|  | P3 – P0 | –2.3±01.0 | 1.5±1.3 | 8.8±6.4 | N/A | –3.7±2.7 | –1.2±1.1 | –2.8±3.3 | –4.8±4.3 |
|  | p–value | .001 | NS | .001 | N/A | .001 | <.001 | NS | .049 |
| D | P1 – P0 | –0.4±0.7 | 7.8±1.6 | 13.7±6.4 | –0.1±0.7 | –6.8±4.5 | –4.5±3.6 | –0.5±2.5 | –3.9±2.6 |
|  | P2 – P0 | –2.2±0.3 | 0.2±0.9 | 13.6±3.5 | 0.9±0.8 | –5.2±4.4 | –4.1±3.7 | –1.8±1.5 | –1.5±2.1 |
|  | P3 – P0 | –2.0±0.1 | –0.3±0.7 | 17.4±5.1 | 3.6±2.6 | –5.8±3.9 | –1.8±3.8 | –2.9±1.7 | 1.4±1.9 |
|  | p–value | <.001 | .003 | .02 | .002 | .004 | <.001 | <.001 | <.001 |
| E | P1 – P0 | –2.4±0.5 | N/A | 28.6±2.0 | 4.1±0.5 | 5.0±4.8 | 0.1±2.1 | 1.8±0.4 | –2.2±5.3 |
|  | P2 – P0 | –2.5±0.6 | N/A | 17.6±2.8 | 2.5±0.9 | 6.8±2.1 | 0.9±2.1 | 0.3±0.8 | –4.8±3.9 |
|  | P3 – P0 | –1.8±0.2 | N/A | 5.5±1.5 | 0.5±21.3 | 6.3±2.4 | 0.2±0.9 | 0.8±1.4 | –4.7±3.7 |
|  | p–value | .01 | N/A | <.001 | .002 | NS | NS | .01 | <.001 |
| F | P1 – P0 | –0.8±0.5 | 3.9±0.9 | 3.7±4.5 | –0.3±0.6 | –0.4±0.9 | –0.8±1.3 | –0.5±1.4 | 0.8±1.6 |
|  | P2 – P0 | –1.0±0.4 | 5.1±1.2 | 1.5±1.5 | –0.5±0.6 | 1.1±0.5 | 15.9±6.8 | 0.1±1.2 | 0.5±1.4 |
|  | P3 – P0 | –0.3±0.3 | 2.6±2.3 | 1.9±1.8 | –0.4±0.7 | –0.7±0.6 | 0.4±0.8 | –0.5±0.8 | 0.0±0.9 |
|  | p–value | <.001 | .03 | .02 | NS | <.001 | <.001 | .02 | NS |
